# Temporal trends of liver cancer burden, comparative analysis of risk factors and trend forecasts to 2024 in China, USA, the Republic of Korea, and Mongolia: an analysis based on multiple data sources from Global Burden of Disease 2019, the Global Cancer Observatory, and Cancer Incidence in Five Continents

**DOI:** 10.7189/jogh.15.04040

**Published:** 2025-01-31

**Authors:** Xing Yao, Xinchun Ling, Ziyi Zhu, Xiaolu Cao, Shaoliang Tang

**Affiliations:** 1 School of Health Economics and Management, Nanjing University of Chinese Medicine, Nanjing, China; 2 School of Pharmacy, Nanjing University of Chinese Medicine, Nanjing, China

## Abstract

**Background:**

Liver cancer represents a significant burden of disease globally, with variations in liver cancer status among countries. In this study, we aimed to evaluate the epidemiological burden of liver cancer in four representative countries – China, the USA, the Republic of Korea, and Mongolia – and cover the highest number of incidence cases, the highest prevalence rates and the burden in developed countries. In addition, we intended to predict the trends in liver cancer in these countries over the next six years.

**Methods:**

We collected epidemiological data on liver cancer from the Global Burden of Disease 2019, the Global Cancer Observatory, and Cancer Incidence in Five Continents databases to conduct data source triangulation. We calculated time trends using Joinpoint regression and predicted incidence rates using an autoregressive integrated moving average model. Aetiological studies were conducted for different countries based on changes in incidence causes.

**Results:**

Between 1990–2019, age-standardised rates (ASR) values for liver cancer declined globally. The downward trend was most pronounced in China, where the average annual percentage change of age-standardised incidence rate (ASIR) reached –3.13 (95% confidence interval (CI) = –2.90, –3.35), much higher than the world average of –1.16 (95% CI = –0.96, –1.36). The ASIR in the USA continued improving and reached 5.23 × 10^5^ in 2019. With age, the ASR for liver cancer in various countries generally shows an upward trend. Hepatitis B virus (HBV) remains the main causative agent of liver cancer in China and Korea. In Mongolia, both HBV and hepatitis C virus account for a large proportion of liver cancer. In the USA, the proportion of liver cancer cases from alcohol consumption has increased annually.

**Conclusions:**

The ASR for liver cancer has declined over the past 30 years in most countries but has worsened in some due to ageing and unhealthy lifestyles.

Primary liver cancer is the sixth most common cancer and the third leading cause of cancer death worldwide in 2020 [1]. It is estimated that there are 9.06 million new cases and 8.30 million deaths worldwide in 2020 [2]. Liver cancer develops mainly from hepatocellular carcinoma cancer (HCC), while the main risk factor for HCC is hepatitis B virus (HBV) or hepatitis C virus (HCV) infection. However, in recent years, excessive alcohol consumption and diseases associated with metabolic syndrome, type 2 diabetes mellitus, obesity, and non-alcoholic fatty liver disease have started to become prominent causes of primary liver cancer as well [3].

Different countries are in different situations, and therefore, their dilemmas are different, and their experiences can be learned from each other. Several countries are special – China, Mongolia, the USA, and South Korea. Of these, China has the largest number of liver cancer cases in the world, currently causing 360 000 cases of morbidity and 350 000 deaths per year in China [4]. Meanwhile, Mongolia is always the first country in the world in terms of liver cancer incidence and mortality. Whereas the USA is representative of the developed world [5], which is experiencing problems that developing countries may be about to face in the future. However, the Republic of Korea is part of the developed world with a more severe burden of liver cancer [6].

Previous epidemiological studies of liver cancer have focused on global trends in liver cancer, with a limited number of country-specific comparative studies. The scarcity of comparative studies may lead to the neglect of country-specific differences and their significance for the development of effective prevention and control strategies. Therefore, this study aims to compare and analyse the burden of liver cancer, its trend, and its risk factors by gender and age group in China, Mongolia, Korea, and the USA by combining several liver cancer-related data sources and to predict further the burden of liver cancer incidence and mortality by 2030. Understanding the risk factors and policies of liver cancer in different countries by comparing the epidemiology of liver cancer in specific countries will help to develop more specific and effective prevention strategies.

## METHODS

### Data sources

In data analysis, the selection of appropriate databases is crucial, and in order to ensure the accurate results of data analysis, we assessed the databases from a variety of aspects such as comprehensiveness, reliability, accessibility, relevance, timeliness, comparability and other considerations. Meanwhile, combining the selection of databases in the existing literature. We selected three authoritative databases as data sources for multi-data source analysis – Global Burden of Disease 2019 (GBD 2019), the Global Cancer Observatory (GLOBOCAN), and Cancer Incidence in Five Continents (CI5). The intention was to carry out data source triangulation in this manner.

The GBD 2019 makes all of its data available to users worldwide, and the types of raw data selected for study in this paper and the processing methods have been set out earlier. It provides a global burden of disease data covering various health indicators and disease types. The comprehensiveness and detail of its data make it an important resource for the study of global health issues. Still, it cannot be ignored that there may be a certain amount of bias in its data, especially if there are inconsistencies in data collection and reporting methods. Further information on the data used in the current study can be found on the GBD Data Entry Source Tool website [7].

GLOBOCAN is a global oncology epidemiology database established by the World Health Organization (WHO). It provides global data related to cancer, focusing on indicators such as cancer incidence, mortality and survival. The specificity and depth of its data make it uniquely valuable in cancer research, but it may lack coverage for certain regions or specific types of cancer. For detailed information about the data used in the current study [8].

CI5 is the world’s definitive compendium of real statistics on tumour epidemiology published by the International Agency for Research on Cancer. Unlike other databases, CI5 provides data on cancer incidence in several countries and regions. Its long history of data accumulation gives it an advantage in trend analyses and historical comparisons. However, the CI5 database is updated slowly, so it is important to be aware that the data may be older, affecting the analyses’ effectiveness.

### Data composition and data filtering

Each of the three data sources has its strengths and weaknesses, so in this article, we present parallel analyses based on the same kind of data from each of them, as well as unique treatments that take advantage of the specific strengths of the different data sources. In order to ensure the quality and validity of the data, the data used for the following analyses have been pre-processed with data cleaning, correction and standardisation.

Based on the data integrity and good timing of the GBD database, we choose to use the GBD database to conduct a time series analysis and forecast the development trend of age-standardised incidence rate (ASIR), age-standardised mortality rate (ASMR), and age-standardised disability-adjusted life rate (ASDR). Further, according to various causes of liver cancer, such as liver cancer (caused by HBV and HCV, etc.), we aimed to analyse the aetiology of liver cancer patients. Since GLOBOCAN focuses more on cancer statistics, and its data comes from the cancer registration system of each country, the data quality is higher, so we chose the liver cancer incidence and mortality data from GLOBOCAN in 2020 to investigate the correlation between the incidence rate and mortality rate of liver cancer and the age of patients.

Finally, we employed data from different cities in CI5, but since the database did not contain information on liver cancer incidence in Mongolia, we focused on three countries, China, South Korea, and the USA, when discussing the differences in liver cancer in different cities. We selected three statistically representative typical cities in each of the three countries according to the gross domestic product (GDP) per capita indicator to represent high, medium, and low levels of economic development, and based on this, we made comparisons of liver cancer incidence rates and other indicators. It is expected that through the analysis of specific cities, the development status of different countries and cities and different strategies for liver cancer treatment and prevention can be combined. Through a comparative study, we aimed to clarify the development status of liver cancer prevention and treatment and explore powerful strategies for liver cancer.

This study is compliant with the Guidelines for Accurate and Transparent Health Estimates Reporting (GATHER) [9].

### Statistical methods

We selected three main databases (GLOBOCAN, GBD 2019, and CI5). We used ASIR, ASMR, and ASDR to estimate differences between geographic areas, historical periods and gender. Further, we calculated the age-standardised rate (ASR): ASIR, and ASDR per 100 000 person-years by summing the age-specific rates (‘αi’ where i denotes the age class) and the number of persons (or weight ‘βi’) in the same age subgroup ‘i’ of the selected reference standard population using the formula [10]:



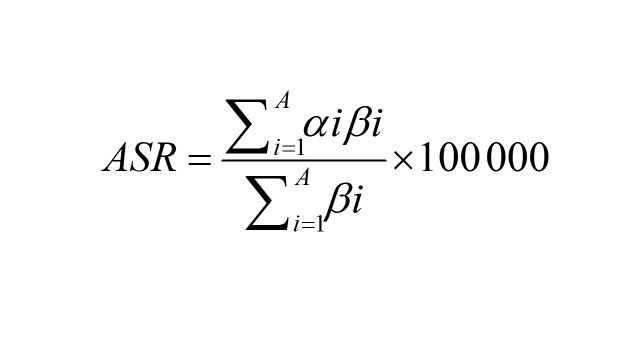



Joinpoint trend analysis uses segmented linear regression to estimate adaptive trends using one or more line segments, and we performed a joinpoint trend analysis using the Joinpoint regression programme, version 5.0.2 (National Cancer Institute, Bethesda, MA, USA) to look at the best-fit annual percentage change (APC) in ASR between 1990–2019 in China, Korea, Mongolia, the USA, and globally. Since the APC index alone is affected by the cancer detection rate, it is suitable for evaluating the internal trend of each independent interval of the segmentation function or the global trend where the number of connecting points is zero. An average annual percentage change (AAPC) is needed when the global average change trend, including multiple intervals, is to be evaluated comprehensively. Therefore, we again used the APC results to weight each interval’s regression coefficients by the width of the span of segmentation intervals. AAPC was obtained to describe the overall time trend in age-standardised rates across countries, with upper and lower confidence limits for each AAPC. 95% uncertainty intervals (UIs) were presented as the 25th and 95th ordered values across all 1000 draws.

We utilised a dual approach incorporating both absolute and relative numeric in analysis. Absolute figures are employed to denote the incidence and mortality rates of liver cancer, underscoring the gravity of its onset. Additionally, we adopted internationally recognised standards such as AAPC and ASIR to maintain academic rigour. In contrast, we used relative figures predominantly in gender-based comparative and aetiological analyses to facilitate comparisons, thereby accentuating the gender disparities in liver cancer prevalence and distinguishing between various pathogenic factors.

Finally, for the different relationships between liver cancer incidence and mortality rates and patients’ age, we selected the most suitable autoregressive integrated moving average model (ARIMA) for each of them. The relationship between liver cancer incidence, mortality rates and age was predicted using a time series model, and we analysed the results as residuals [11]. Based on the computation of the indicators above, we made the decision to utilise the ARIMA model for the projection of incidence rates associated with liver cancer. The ARIMA model is a statistical model specifically designed for time series forecasting. The ARIMA model assumes that the trend of time series data are characterised by smoothness and that the covariance between random variables at any two time points is only related to the time interval between them. It also assumes that the value at the current time point is influenced by the values at the past number of time points and that there is a dependency relationship. At the same time, the prediction error will also be affected by the prediction errors of the past several time points. For the unsteady series, it can be eliminated by first-order or multi-order differencing to eliminate the trend factor and seasonal factor of the series so that the series meets the assumption of smoothness. The residual terms of the model should satisfy the independence assumption. We implemented the main statistical analyses and graphics in the current study using Python, version 4.0.3 (Python Software Foundation, Wilmington, DE, USA). We considered a two-sided *P*-value of <0.05 statistically significant [11].

## RESULTS

### Temporal trends in liver cancer burden

**Figure 1:**
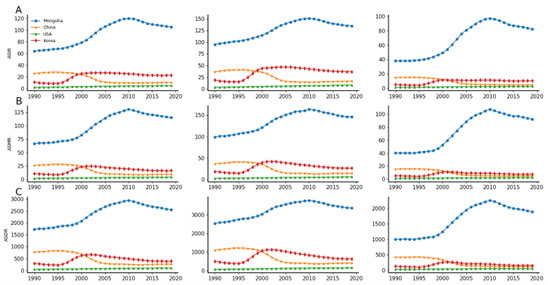
Trends for ASR of liver cancer in China, the USA, the Republic of Korea, Mongolia from 1990 to 2019 by sexes. **Panel A.** ASIR (per 100 000 population). **Panel B.** ASMR (per 100 000 population). **Panel C.** ASDR (per 100 000 population). From left to right, the indicators of all personnel, including men and women, are indicated. ASIR – age-standardised incidence rate, ASMR – age-standardised mortality rate, ASDR – age-standardised disability-adjusted life-year rate.

We analysed time trends in liver cancer burden in China, the USA, the Republic of Korea, Mongolia, and the world using data from GBD 2019. Table S1 in the **Online Supplementary Document** demonstrates the mortality trend of liver cancer cases in China, the USA, Mongolia, and the Republic of Korea. The global ASIR for liver cancer declined from 1990 to 2019 (AAPC = –1.16; 95% CI = –0.96, –1.36). China’s downward trend over these 30-year periods was greater than the global average (AAPC = –3.13; 95% CI = –2.90, –3.35). In 2019, there were 210 462.35 new cases of liver cancer in China, with an ASIR of 10.46 per 100 000 population and an ASDR of 2558.12 per 100 000 population. Compared to other countries, Mongolia’s liver cancer burden has been high since 1990, with the highest ASMR value of 130.68 per 100 000 population (95% UI = 109.97, 153.76), which far exceeds the burden status of other countries. Korea also experienced a rapid increase in ASMR values between 1995–2000, which led it to surpass China’s 23.91 per 100 000 population (95% UI = 21.74, 26.19) in 2000, making it the country with the second highest burden of liver cancer among these four countries. However, in the last decade, the burden of liver cancer in China, the Republic of Korea, and Mongolia have begun to gradually decline or level off. As a representative of many developed countries, the burden level of liver cancer in the USA has always been lower than that in the other three countries, but it has maintained a continuous growth during these 30 years, and its AAPC value of ASDR for liver cancer has even come to 2.58 (95% CI = 2.64, 2.51), which is much higher than the international average (**Figure 1**).

In terms of gender, 70.45% of liver cancer cases globally occurred among men in 2019, a slight decrease from 69.07% in 1990. The proportion of male liver cancer patients in China (75.92%), the USA (72.18%), and the Republic of Korea (74.61%) has remained stable at around 70%. The difference is that the proportion of Mongolian male liver cancer patients decreased from 68.21% to 59.09% (incidence = 1.37 × 10^3^). Between 1990–2010, the ASIR value for liver cancer in Mongolian males only improved by about half, while the ASIR value for Mongolian females increased by almost 1.5 times, which significantly narrowed the difference in the burden of liver cancer between Mongolian males and females (**Figure 1**). It is important to note that, unlike the narrowing of the gender difference in liver cancer in Mongolia, the gender difference in liver cancer burden in China has remained at a high level and has now overtaken Mongolia as the most severe of the four countries (ASIR male to female ratio of 3.15).

### Age composition of liver cancer burden

**Figure 2:**
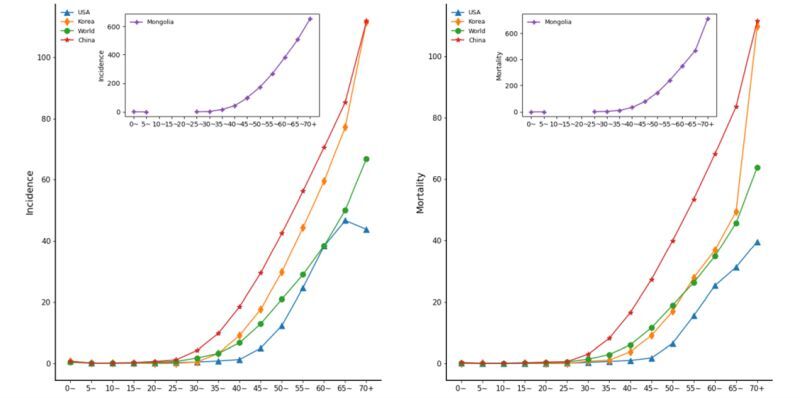
Trend of age-specific liver cancer burden in China, the USA, the Republic of Korea, Mongolia, and the world in 2020. **Panel A.** ASIR (per 100 000 population. **Panel B.** ASMR (per 100 000 population). ASIR – age-standardised incidence rate, ASMR – age-standardised mortality rate.

Using GLOBOCAN 2020 data to study the age composition of liver cancer, we found that the incidence and mortality of liver cancer varied considerably with age (**Figure 2**). The overall trend is a sharp increase starting after the age of 25 years, with the growth rate increasing each year, reaching the country’s ASIR for the whole year in the age group of 40–45 years.

Among the four countries, Mongolia continues to show the most significant growth trend. In Mongolia, the incidence and mortality rates for people aged >70 years exceeded 600 per 100 000 population, while the world average is only 65.5 per 100 000 population and 62 per 100 000 population. The incidence and mortality trends by age are similar in all countries. Still, unlike other countries that have continued to grow, the peak incidence of liver cancer in the USA in 2020 is in the age group of 65–70 years, reaching 46.7 per 100 000 population. Meanwhile, of the four countries, only the USA has incidence and mortality values lower than the world average for all ages. It is worth noting that the incidence rate of liver cancer in Korea at all ages is similar to that of China and slightly higher than the world average. However, the mortality rate of liver cancer in people aged <65 years is consistently low, similar to the world average and much lower than that of China.

### Aetiological analysis of liver cancer burden

For the Republic of Korea and China, their burden of liver cancer is mainly caused by HBV (Figure S1 in the **Online Supplementary Document**). This is especially true for men in China, where the percentage of liver cancer caused by HBV in Chinese men was consistently higher than 75% during these 30-year periods. In the USA, on the other hand, the contribution of HBV to the incidence of liver cancer is relatively small (male: 15.1%, female: 11.83%), replaced by other risk factors such as alcohol consumption and HCV.

In 2019, liver cancer caused by alcohol consumption accounted for 38.97% of all cases in men in the USA, and HCV caused 30.19% of all liver cancer cases. In Mongolia, the country with the highest burden of liver cancer, HBV, HCV, and alcohol consumption contributes similarly to the disease burden. In 2019, 34.76% of liver cancers in Mongolian males were caused by HBV, HCV determined 20.77%, and 37.95% were due to the habit of alcohol consumption. The situation was similar for Mongolian females. 22.68% were caused by HBV, 20.61% were determined by alcohol consumption, and 41.92% were determined by HCV.

While the contribution of HBV to the burden of liver cancer in each country has gradually declined over time, the share of liver cancer caused by alcohol consumption has increased, especially in Mongolia, where the share of liver cancer caused by alcohol consumption in males increased from 30.76% in 1990 to 37.95% in 2019.

When observed from a gender perspective, the etiological differences in liver cancer burden between males and females are large. Compared to males, females have a higher percentage of liver cancer caused by HCV, non-alcoholic steatohepatitis (NASH), or other causes. Especially in the Republic of Korea, the percentage of liver cancer caused by HCV reached 34.99% in females in 2019, far exceeding the 8.78% in males. In China, the USA, and the Republic of Korea, the number of women with liver cancer caused by NASH is close to the number of women with liver cancer caused by alcohol consumption, all remaining at around 10%.

### Urban comparison of crude and standardised liver cancer rates

**Figure 3:**
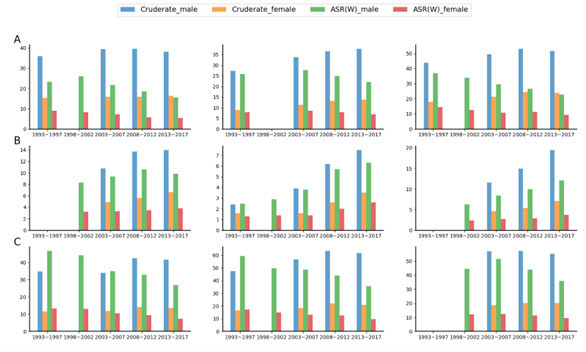
Crude and world standard rates of liver cancer in China, the USA, and the Republic of Korea by city, 1993–2017. **Panel A.** China (left: Shanghai, middle: Wuhan, right: Jiashan). **Panel B.** USA (left: Los Angeles, middle: Utah, right: New Mexico). **Panel C.** The Republic of Korea (left: Seoul, middle: Pusan, right: Jeju Island).

Based on the GDP metrics of each region, three representative regions in each of China, the USA, and the Republic of Korea included in the CI5 database were selected for incidence analysis (**Figure 3**). Between 1993–2017, China, the USA, and the Republic of Korea each had a distinctive incidence profile.

The CI5 incidence long-term trend showed that the crude rates of each representative city in China and Korea are in a slight fluctuation or a slow increase, but their ASR values have all remained almost steadily decreasing. Shanghai, in particular, saw a 6% increase in the crude incidence rate for men and a 7% increase in the crude incidence rate for women between 2013–17 timeframe compared to the timeframe between 1993–97, but their ASIR of liver cancer decreased by 33.40% and 41.10%, respectively. The representative regions of the USA – Los Angeles, Utah, and New Mexico – maintained increasing incidence and ASIR over these 24-year periods, consistent with the overall liver cancer trends in the USA described earlier. The difference in liver cancer burden between cities within the same country is relatively small compared to the disparity between countries.

### Liver cancer incidence forecasts for China, the USA, the Republic of Korea, and Mongolia

We used the GBD database data from 1990 to 2019 to predict the incidence of liver cancer in China, the USA, the Republic of Korea, and Mongolia and to give targeted recommendations. The prediction results show that the ASIR in China will fluctuate slightly from 10.50 per 100 000 population to 10.53 per 100 000 population by 2024. On the other hand, the ASIR values of the USA and the Republic of Korea showed a significant increase from 5.23 per 100 000 population and 22.67 per 100 000 population in 2019 to 5.68 per 100 000 population and 25.20 per 100 000 population in 2024, an increase of 9% and 11%, respectively (Table S2 in the **Online Supplementary Document**). Notably, the ASIR of Korean males will decrease from 36.96 per 100 000 population to 36.46 per 100 000 population, contrary to the overall trend.

Contrary to China, the USA, and the Republic of Korea, the ASIR in Mongolia will remain in decline between 2019–24. It is expected to decrease from 105.35 per 100 000 population to 96.41 per 100 000 population over the next five years, a decrease of 8.49%.

## DISCUSSION

In the research of the temporal trends in liver cancer spanning three decades, we observed a marginal improvement in the global burden of liver cancer in the last decade. However, it continues to significantly impact populations in the USA, South Korea, and Mongolia. The ASR values in Mongolia remain alarmingly high, with the ASDR reaching 2558.11 per 100 000 population in 2019, a 17 times higher than the global average. This is particularly relevant to irregular access restrictions for medical injections and other health care-related procedures in Mongolia, a primary cause of HCV infection [12]. Conversely, the USA, which has maintained a low count of HBV patients due to extensive HBV vaccine coverage, is witnessing an upward trend in liver cancer as the burden escalates with an ageing population [13]. It is imperative that public health policies and interventions are devised and executed to mitigate the disproportionately high mortality from HCC in the USA [13].

HBV is a predominant cause of liver cancer in China and Korea [14]. It is of significant note that despite the rapid population growth in China, the incidence of liver cancer has seen a decline, resulting in fewer cases in 2019 than in 1990. This can be largely attributed to the proactive interventions against HBV by the Chinese government [15]. As highlighted by Cao et al., the number of HBV patients in China has seen a dramatic decrease, a consequence of a multitude of policies and related laws enacted by the Chinese government since 2005 [13].On the other hand, South Korea initiated immunisation against HBV in the 1990s, which has led to a certain degree of reduction in the incidence of liver cancer. However, the burden of liver cancer in South Korea continues to escalate, a trend that can be associated with the dietary habits of the Korean population, particularly the consumption of kimchi [16].

In the analysis of gender-specific results, we found that the global burden of liver cancer in males still exceeds that in females by more than 2-fold, a relationship closely linked to the protective effects of female sex hormones [17]. Additionally, in China, Liu et al. discovered that the accumulation of risk factors such as alcohol consumption and insomnia contributes to male HBV patients being more prone to developing cirrhosis or liver cancer than females [18]. It is noteworthy that the burden of liver cancer in Mongolian women has rapidly increased since 1990, with an AAPC reaching 2.68, which is 2.23 times that of Mongolian men. This trend may be associated with changes in Mongolia’s gender structure [19]. In recent years, the female population in Mongolia has grown rapidly. Simultaneously, due to the significantly more severe alcohol consumption among Mongolian women compared to women in other countries, the proportion of female liver cancer patients caused by alcohol in Mongolia is much larger than in other nations.

In addition to risk factors such as HBV and alcohol consumption, we also explored the aetiology of liver cancer, including various factors such as HCV and NASH, and analysed their impact on different gender populations in four representative countries [20]. There are significant differences in the distribution of liver cancer risk factors among different countries [21]. In China and the Republic of Korea, HBV remains the predominant risk factor, especially for males. In Mongolia, which has the highest global burden of liver cancer, HCV is also a significant factor. Over the period between 1990–2019, the number of liver cancer cases in Mongolian women attributed to liver HCV has consistently hovered around 42%. Regarding the prevalence of HBV and HCV in Mongolia, Znaor et al. attributed it to the insufficient coverage of hepatitis vaccination for newborns and children, as a modelling study indicated a decreasing HBV seroprevalence only in cohorts born after 1995. In addition, the incidence of liver cancer in Mongolia can also be attributed to the prevalent drinking habits. It is known that within the country, 50% of males and 27% of females are reported to be heavy drinkers [22]. It is noteworthy that, in recent years, the proportion of liver cancer caused by alcohol consumption in the USA has been steadily increasing, accompanied by a continuous rise in liver cancer ASIR, ASMR, and ASDR. In addition to the mentioned risk factors, liver cancer has been proven to be closely associated with factors such as aflatoxin, high iron food and tobacco intake [23,24]. Ming et al. observed through a follow-up study that aflatoxin exposure triples the risk of liver cell carcinoma in males with HBV infection, and approximately 4.60–28.20% of liver cell carcinoma worldwide is caused by aflatoxin consumption [25].

The results of the comparative analyses of representative cities demonstrate that the differences between different countries are greater than those between different cities in the same country. There are some differences among cities in the same country, but the overall level is similar, which indicates that the correlation between the urban GDP index and the burden of liver cancer is not strong. We speculate that the living environment, cultural customs and other social factors in a region are the important reasons for the differences in the burden of liver cancer among different cities. Qidong city is a good example, where molecular epidemiological and genome deep sequencing studies have confirmed that HBV infection, the use of aflatoxin-contaminated maize as a staple food and the direct consumption of well water containing high levels of microcystin may be important aetiological factors contributing to the high incidence of liver cancer in the region [26,27]. Chen and Kensler also showed in their study that the city has, in the last few decades, been ageing, and ageing is the greatest predictor of most types of cancer [27]. Thus, it is clear that a variety of factors influence the incidence of liver cancer and cannot be described by a single economic or other indicator.

In the age-structure study, we found a strong positive correlation between liver cancer incidence and mortality in all countries. Petrick et al. confirmed that in most countries, the peak incidence of liver cancer occurs around 75 years old [28]. It is worth noting that when analysing different cities using CI5, we found that the crude rate increased in some cities during the 24 years while the standardised rate decreased continuously. This situation is most evident in China and Korea, which is due to the increasing ageing procedures in some cities of these two countries [29]. In Shanghai, China, for example, the population aged ≥60 years in 2020 was 5.82 million, or 23.40%, an increase of 8.30% points over the decade [30]. The change in the Shanghai population is reflected in the liver cancer data in CI5. Between 1993–2017, the ASIR of liver cancer in Shanghai women decreased by 41.11%, but the crude incidence increased by 7.19%. It can be seen that the growing elderly population is creating a new wave of impact on the burden of liver cancer in all countries.

The effects of this ageing are also reflected in our projections. In the forecast results for the next five years between 2020–24, we find that, unlike the rapid decline between 2000–10, China’s ASIR will increase from 10.50 per 100 000 population to 10.53 per 100 000 population, and the Republic of Korea’s ASIR value will also increase from 22.67 per 100 000 population in 2019 to 25.20 per 100 000 population in 2024. Notably, Mongolia shows a rapid downward trend in the next five years. We speculate that this is related to the fact that with the improvement of medical awareness in Mongolia in recent years, the reuse of syringes has been significantly improved, and the disinfection of medical equipment has gradually received attention [12]. Simultaneously, multiple health organisations have proposed corresponding plans and provided economic and technical assistance to Mongolia, significantly increasing the prevalence of hepatitis vaccination in the country [22].

This study has two main advantages. First, combining multiple data analyses from multiple databases allows for a broader study coverage of comparative analyses of liver cancer and cuts down on the impact of statistical errors in a single database on the findings. It will also provide new perspectives on understanding global and regional differences in liver cancer. Second, the selection of representative countries and cities for comparative studies allows for more in-depth mining and analysis of the data, including exploring differences between regions within countries, leading to more nuanced insights.

However, this research still has some limitations. First, the data on liver cancer in the GBD database lacks information on histopathological types and clinical stages. However, this information is useful for understanding the severity of the disease and providing deeper insights into disease prognosis and treatment modalities. The absence of such information may preclude the conduct of precision research and the formulation of targeted health care strategies, thereby affecting the applicability of our findings in clinical settings. Second, the accuracy of our research results is contingent upon the accuracy of the database. Nevertheless, we cannot circumvent the potential for data distortion arising from variations in measurement practices across different countries and regions, as well as from errors in statistical processes, which may introduce bias into our results. Lastly, given the numerous factors influencing hepatocellular carcinoma, such as alcohol consumption, genetics, radiation exposure, and chemical substances, the study could not exhaustively address all possible factors. This omission may result in an incomplete analysis of the disease aetiology, thus impacting the development and efficacy of disease prevention strategies.

## CONCLUSIONS

In conclusion, the burden of liver cancer in most countries in the world has improved greatly in the past decade. However, the developed countries represented by the USA have maintained a rising trend year by year. Due to the aggravation of the ageing population in China, the Republic of Korea, and other Asian countries, the reduction rate of the burden of liver cancer is slowing down year by year. Therefore, countries need to curb the major causes of liver cancer in their countries, such as Korea and China, which are ageing urban countries, should increase the surveillance of liver cancer in the elderly population. In developed countries, the public needs to be encouraged to adopt healthy lifestyles, such as reducing alcohol consumption, eating a sensible diet, and increasing physical activity, in order to prevent NASH and related liver cancers. Developing countries such as Mongolia still need to increase support and access to vaccines, as well as cancer screening for people who drink alcohol.

## Additional material


Online Supplementary Document
