# Supplementary material for: Temporal trends of liver cancer burden, comparative analysis of risk factors and trend forecasts to 2024 in China, USA, the Republic of Korea, and Mongolia: an analysis based on multiple data sources from Global Burden of Disease 2019, the Global Cancer Observatory, and Cancer Incidence in Five Continents
Source: J Glob Health. 2025 Jan 31;15:04040. doi: 10.7189/jogh.15.04040 (PMC11783136; doi:10.7189/jogh.15.04040)
Supplement: Online Supplementary Document [file jogh-15-04040-s001.pdf]

Supplement to: Yao X, Ling X, Zhu Z, Cao X, Tang S. Temporal trends of liver cancer burden, comparative analysis of risk factors and trend forecasts to 2024 in China, USA, Republic of Korea, and Mongolia – analysis based on multiple data sources from Global Burden of Disease 2019, the Global Cancer Observatory, and Cancer Incidence in Five Continents. J Glob Health 2025;15:04040.

Table S1. The number, age-standardized rate, and changing trend of liver cancer burden in China, the USA, the Republic of Korea, Mongolia and the world from 1990 to 2019, by sexes 1

Table S2. Four-Country Five-Year Incidence Rate Forecast 4

Figure S1. Percentage change in causes of liver cancer by gender in four countries from 2019 to 2020((Male: left, Female: right).Panel A. China; Panel B. United States; Panel C. The Republic of Korea; Panel D. Mongolia) 5

Table S1. The number, age-standardized rate, and changing trend of liver cancer burden in China, the USA, the Republic of Korea, Mongolia and the world from 1990 to 2019, by sexes

	ASIR					ASMR					ASDR				
	1990		2019		1990-2019	1990		2019		1990-2019	1990		2019		1990-2019
	No.* 10^4	ASR (per 100000 population)	No.* 10^4	ASR (per 100000 population)	AAPC (95%CI)	No.* 10^4	ASR (per 100000 population)	No.* 10^4	ASR (per 100000 population)	AAPC (95%CI)	No.* 10^4	ASR (per 100000 population)	No.* 10^4	ASR (per 100000 population)	AAPC (95%CI)
Both															
China	23.68	25.71	21.04	10.46	-3.13 (-2.90,-3.35)	23.24	25.99	18.76	9.41	-3.42(-3.12,-3.72)	757.78	769.11	532.55	264.31	-3.60(-3.31,-3.88)
Mongolia	0.07	64.22	0.23	105.22	1.72 (1.82.1.62)	0.07	66.77	0.24	115.23	1.86 (2.05,1.66)	1.91	1726.2	6.5	2558.12	1.39(1.55,1.22)
USA	0.69	2.22	2.78	5.23	2.98 (3.04,2.91)	0.65	2.04	2.38	4.33	2.64 (2.70,2.57)	15.23	51.35	55.13	107.18	2.58(2.64,2.51)
Republic of Korea	0.35	10.99	2.03	22.8	2.60 (3.00,2.19)	0.34	11.04	1.45	16.2	1.32 (1.64,0.99)	10.54	297.39	34.8	390.81	0.97(1.30,0.65)
Global	37.34	8.98	53.43	6.51	-1.16 (-0.96,-1.36)	36.52	8.93	48.46	5.95	-2.16(-1.53,-2.78)	1127.86	258.37	1252.84	151.08	-2.33(-1.50,-3.15)
Males															

	ASIR					ASMR					ASDR				
	1990		2019		1990-2019	1990		2019		1990-2019	1990		2019		1990-2019
	No.* 10^4	ASR (per 100000 population)	No.* 10^4	ASR (per 100000 population)	AAPC (95%CI)	No.* 10^4	ASR (per 100000 population)	No.* 10^4	ASR (per 100000 population)	AAPC (95%CI)	No.* 10^4	ASR (per 100000 population)	No.* 10^4	ASR (per 100000 population)	AAPC (95%CI)
China	17.01	36.44	15.97	16.36	-2.79 (-2.54,-3.03)	16.49	36.65	13.9	14.56	-3.09(-2.74,-3.44)	559.04	1102.84	414.57	414.9	-3.29(-2.96,-3.62)
Mongolia	0.05	95.05	0.13	134.83	1.20 (1.31,1.07)	0.05	99.22	0.13	146.03	1.30 (1.50,1.10)	1.33	2540.12	4.04	3374.04	0.98(1.14,0.82)
USA	0.44	3.27	2.01	8.09	3.12 (3.16,3.07)	0.4	2.95	1.63	6.49	2.71 (2.79,2.64)	9.88	73.92	39.65	162.25	2.72(2.79,2.64)
Republic of Korea	0.26	18.77	1.51	36.82	2.42 (2.80,2.04)	0.25	18.99	1.06	26.32	1.11 (1.33,0.90)	8.12	501.3	27.38	638.94	0.88(1.13,0.62)
Global	26.02	13.07	37.64	9.71	-1.09 (-0.97,-1.20)	25.1	12.9	33.36	8.73	-2.07(-1.40,-2.77)	805.88	378.19	904.87	225.28	-2.28(-1.42,-3.14)
Females															
China	6.67	14.97	5.07	4.94	-3.82 (-3.52,-4.12)	6.75	15.57	4.87	4.76	-3.09(-2.74,-3.44)	198.74	420.06	117.97	115.85	-4.32(-4.12,-4.53)
Mongolia	0.02	38.19	0.09	82.37	2.68 (3.10,2.27)	0.02	40.2	0.1	92.08	2.85 (3.12,2.59)	0.58	997.89	2.46	1888.24	2.16(2.50,1.82)

	ASIR					ASMR					ASDR				
	1990		2019		1990-2019	1990		2019		1990-2019	1990		2019		1990-2019
	No.* 10^4	ASR (per 100000 population)	No.* 10^4	ASR (per 100000 population)	AAPC (95%CI)	No.* 10^4	ASR (per 100000 population)	No.* 10^4	ASR (per 100000 population)	AAPC (95%CI)	No.* 10^4	ASR (per 100000 population)	No.* 10^4	ASR (per 100000 population)	AAPC (95%CI)
USA	0.25	1.38	0.78	2.67	2.27 (2.35,2.20)	0.25	1.33	0.74	2.43	2.10 (2.24,1.96)	5.35	32.42	15.47	56.87	1.96(2.11,1.81)
Republic of Korea	0.09	5.27	0.52	10.55	2.14 (2.83,1,45)	0.09	5.45	0.38	7.63	1.02 (1.60,0.44)	2.42	130.99	7.42	157.61	0.33(1.27,-0.60)
Global	11.32	5.22	15.79	3.63	-1.32 (-1.21,-1.42)	11.42	5.33	15.09	3.46	-2.02(-1.53,-2.51)	321.99	143.38	347.97	81.27	-2.05(-1.31,-2.78)

ASIR - age-standardized incidence ratios, ASMR - age-standardized mortality ratio, ASDR - age-standardized mortality rates, No. - Number of population, ASR(per 100000 population) - age-standardized incidence rates per 100000 population.

Table S2. Four-Country Five-Year Incidence Rate Forecast

	Predict incidence rate Both	Predict incidence rate Fmale	Predict incidence rate Male
**China**			
2020	10.49142651	4.896287354	16.40349474
2021	10.50319079	4.843659166	16.41302353
2022	10.51495507	4.785900969	16.4068505
2023	10.52671935	4.725869982	16.39379651
2024	10.53848363	4.664832061	16.37772709
**USA**			
2020	5.31790136	2.67205933	8.268863154
2021	5.408370632	2.678692672	8.446087189
2022	5.498839904	2.685344402	8.623511301
2023	5.589309176	2.692000622	8.80088621
2024	5.679778448	2.698657936	8.978273218
**South Korea**			
2020	23.20497599	10.70804968	36.77649962
2021	23.82170326	10.85893161	36.70914417
2022	24.45220081	11.00752783	36.63036739
2023	24.8815042	11.1551496	36.5468071
2024	25.20337386	11.30235591	36.46124335
**Mongolia**			
2020	103.6896394	80.56057954	133.9973361
2021	101.9524745	78.60642572	133.1424644
2022	100.0361256	76.5733128	132.2792058
2023	98.18935958	74.49678808	131.4131101
2024	96.40612019	72.3963955	130.5460547

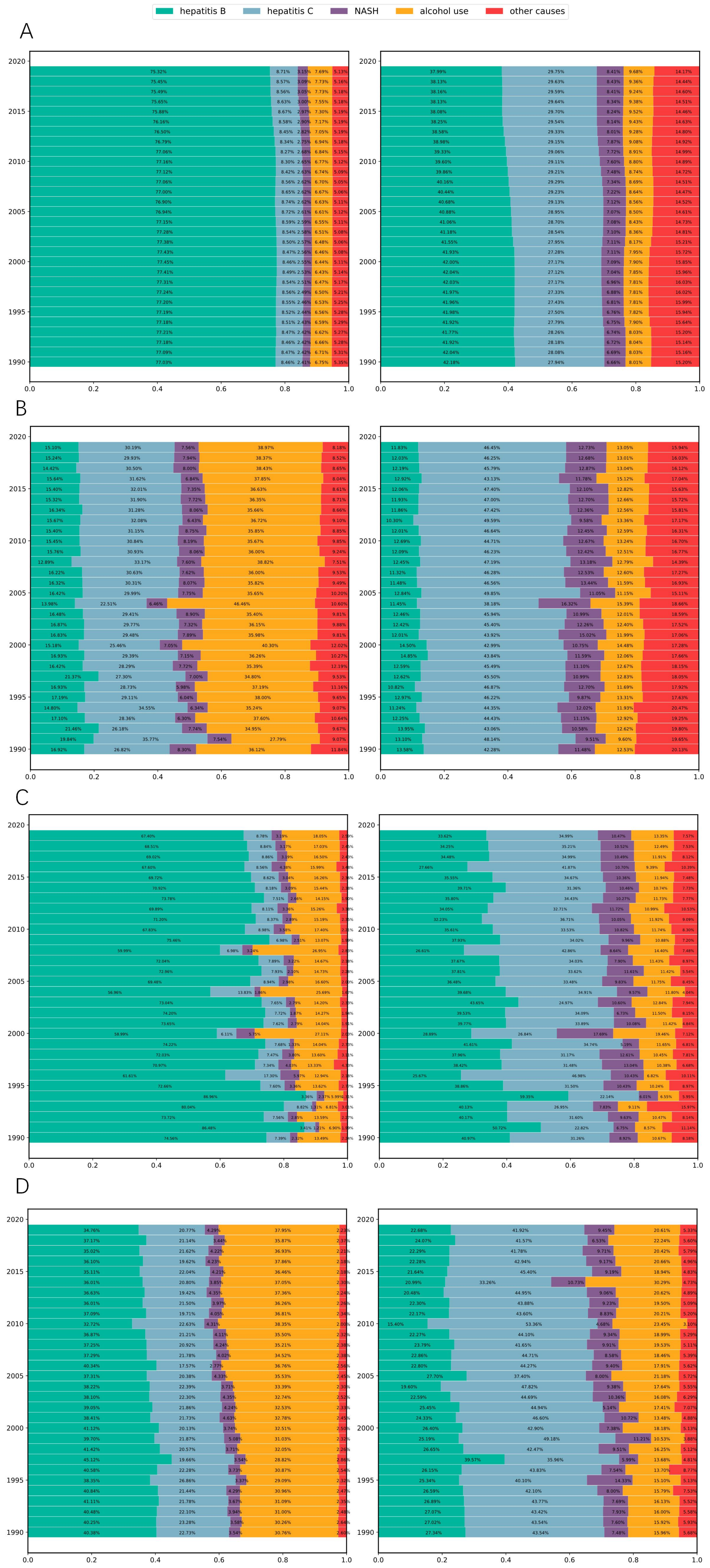

Figure S1. Percentage change in causes of liver cancer by gender in four countries from 2019 to 2020((Male: **left**, Female: **right**).**Panel A.** China; **Panel B.** United States; **Panel C.** The Republic of Korea; **Panel D.** Mongolia)
